# Land ownership and technology adoption revisited: Improved maize varieties in Ethiopia

**DOI:** 10.1016/j.landusepol.2017.12.047

**Published:** 2018-03

**Authors:** Di Zeng, Jeffrey Alwang, George Norton, Moti Jaleta, Bekele Shiferaw, Chilot Yirga

**Affiliations:** a Centre for Global Food and Resources, 6.24 Nexus 10, University of Adelaide, Adelaide, SA 5005, Australia; b Department of Agricultural and Applied Economics, 215I Hutcheson Hall, Virginia Tech, Blacksburg, VA 24061, United States; c Department of Agricultural and Applied Economics, 205B Hutcheson Hall, Virginia Tech, Blacksburg, VA 24061, United States; d International Maize and Wheat Improvement Center (CIMMYT), P.O. Box 5689, Addis Ababa, Ethiopia; e World Bank Group, 1818 H Street NW, Washington DC 20433, United States; f Ethiopian Institute of Agricultural Research (EIAR), P.O. Box 2003, Addis Ababa, Ethiopia

**Keywords:** Land ownership, Technology adoption, Maize, Improved varieties, Land contract, Ethiopia

## Abstract

The lack of land ownership can discourage agricultural technology adoption, yet there is scarce evidence of the impact of land rental contracts on the adoption of improved crop varieties in developing countries. The current study investigates such impact using a nationally representative survey of Ethiopian maize farmers. In contrast to many previous studies, we show in a simple model that cash-renters are as likely to adopt improved maize varieties as owner-operators, while sharecroppers are more likely to adopt given that such varieties are profitable. Empirical analysis reveals a significant impact of sharecropping on improved maize variety adoption, and no significant impact from cash-rental, lending support to the above hypotheses. These results imply that improvements in land rental markets can potentially enhance household welfare through crop variety adoption in agrarian economies where land sales markets are incomplete or missing.

## Highlights

• We study whether land rental discourages improved crop variety adoption.
• We show in theory that cash-renters are indifferent, but sharecroppers are encouraged.
• Empirical results from Ethiopian maize farmers support the above hypotheses.
• Results imply improvements in land rental markets can improve farmers’ welfare.

## 1 Introduction

Land ownership, or land tenure, has been increasingly investigated as a factor affecting modern agricultural technology adoption in Sub-Sahara Africa (SSA). From both theoretical and empirical perspectives, Gavian and Fafchamps (1996) find secure tenure encourages investments in soil conservation technologies in northern Ethiopia. Abdulai et al. (2011) conclude that land ownership tends to facilitate investment in soil-improving and natural resource management practices in Ghana. Oostendorp and Zaal (2012) also suggest that transfer rights, a measure of land ownership, stimulate the adoption of soil and water conservation technologies in Kenya. It is generally hypothesized that land ownership encourages agricultural technology adoption, while the lack of land ownership discourages it. The underlying argument is that the lack of landownership, as usually reflected in land rentals, may preclude tenants from future technology-induced benefits due to the risk of eviction. Land ownership, on the contrary, can safeguard cash flows over time and facilitate asset liquidation given transferrable land rights and can also enhance access to resources such as credit (Feder and Nishio, 1998). All these factors can incentivize the adoption of technologies that require investments and that potentially increase the value of land.

Empirical findings of this literature, however, are mixed the hypothesized impacts can sometimes bear opposite signs and their magnitudes are usually small (see Brasselle et al., 2002; Place, 2009; Fenske, 2011 for literature syntheses from different perspectives). Such inconclusiveness is partly due to the failures to differentiate the varying characteristics of agricultural technologies. For example, Deininger and Jin (2006) show that the lack of land ownership, or tenure insecurity, can either discourage agricultural technology investment (if ownership security is exogenous) or encourage investment (if ownership security is endogenous). The latter observation accords with earlier literature that the threat of non-renewal may cause tenants to work harder and produce more (Cheung, 1969). Place (2009) further shows that the divergent impacts of land ownership on the adoption of different technologies in a comprehensive literature review. Hence, characteristics of agricultural technologies need careful differentiation to help disentangle any confounding impacts in search of policy implications.

While most studies in this literature focus on resource-conserving technologies, modern agricultural technologies also include productivity-enhancing ones such as improved crop varieties and fertilizer (Ersado et al., 2004), and possible impacts of land ownership on the latter need to be better understood. This literature bias could be partly driven by the belief that land ownership affects only long-term investments related to natural resource management but not short-term input use decisions. However, this is not generally true because even seasonal crop variety choices may have deferred impacts on productivity or risk-mitigation that could affect investments, which in turn depends on land ownership. Although a few recent studies have analyzed the impact of land ownership on the adoption of fertilizer (e.g. Abdulai et al., 2011; Ali et al., 2012), these studies fail to consider crop variety choices which potentially affect fertilizer application decisions (Heisey and Norton, 2007). Despite land market imperfections, improved crop varieties have been a major driver of agricultural productivity growth in SSA (see Evenson and Gollin, 2003 for a comprehensive cross-country analysis), which further results in welfare improvements in terms of poverty reduction (Kassie et al., 2011; Zeng et al., 2015), and food security (Shiferaw et al., 2014). Understanding how land ownership affects improved crop variety adoption is therefore highly relevant in assisting ongoing market-oriented land reforms in SSA.

Empirical identification of the hypothesized impact is difficult due to confounding effects. For instance, resource-conserving practices such as tree planting can be adopted to demonstrate and strengthen claims to land rights (Place and Otsuka, 2002), while productivity-enhancing practices such as organic fertilizer that improves soil capital can also be adopted by tenants to increase the chance to continue land operation in the future (Abdulai et al., 2011). In both cases, causality can be reverse, but such potential endogeneity is not commonly recognized (Brasselle et al., 2002; Fenske, 2011). Moreover, although the lack of land ownership is mainly manifested through land rental contracts,^1^ few studies differentiate contract types such as cash-rental or sharecropping due to data limitations. These complexities need to be clearly understood by policy makers who hope to improve rural welfare from this perspective.

The current study assesses the impacts of land rental, as associated with two most important land rental contracts (cash rental and sharecropping), on improved crop variety adoption using a nationally representative survey of maize farmers in Ethiopia. We show in a simple model that, unlike the case of resource-conserving technologies, land rental does not discourage the adoption of improved crop varieties for cash-renters, but encourages adoption for sharecroppers if such varieties are profitable. Empirical evidence is robust in support of these hypotheses, suggesting that improvements in land rental markets can potentially enhance household welfare in agrarian economies where land sales markets are incomplete or missing.

## 2 Land ownership and maize production in Ethiopia

Land tenancy in Ethiopia has a long history, which stems from the feudal system that existed before the Derg government took power in 1974. Land distribution was skewed and a large share of land was operated by tenants. Early literature shows that the share of rented land was over 40 percent, and operating tenants represented a similar proportion of the total population (Rahmato, 1984). Sharecropping was the dominant type of land rental (Holden et al., 2008).

Land rental has been present in Ethiopia throughout history. Arbitrary eviction of tenants was a major feature of the land rental system in the feudal society (Deininger and Jin, 2006). The land reform in 1975 confiscated all land as state property, and cultivators were left with only user rights but prohibited from land rentals and labor hiring (Holden et al., 2008). Further, land redistribution through governmental power was common during the Derg regime under the stated objectives of overcoming inequality and landlessness (Fenske, 2011). Since the current government took power in 1991, land redistributions were largely reduced (with the exception of land redistribution in Amhara region in 1997–1998) and short-term land renting and labor hiring were legalized. However, as permanent land transfer continues to be prohibited by enforced policies, the land sales market is still nonexistent in rural Ethiopia. Land inheritance is allowed and creates incentives for land rentals (Crewett and Korf, 2008). As a result, the short-term land rental market is expanding, and plot rentals are common due to land fragmentation (Benin et al., 2005). The scenario of Ethiopia therefore provides a unique context of study as the land rental market plays an active role to meet the expanding land demand without land sales market, and possible policy implications of the current study may also apply to other agrarian economies in SSA where land sales markets are underdeveloped and land rental widely exists.

Maize is one of the most important food and cash crops in SSA. In Ethiopia, maize accounts for the largest share of production by volume and is produced by more farmers than any other crop (Chamberlin and Schmidt, 2012). During the 2009–2010 production year, Ethiopia produced 3.89 million tons of maize on 1.77 million hectares of land (Central Statistical Agency, 2010). The average productivity of 2.20 tons per hectare was the highest among all cereal crops in the country.

In the last four decades, more than 40 improved maize varieties have been developed through joint efforts of the Ethiopian Institute of Agricultural Research and the International Maize and Wheat Improvement Center (CIMMYT). Improved maize seeds have been diffused mainly through the Ethiopian Seed Enterprise, the major seed producer and distributor, while regional seed enterprises such as Oromia Seed Enterprise, Amhara Seed Enterprise, and Southern Seed Enterprise also produce and sell maize seeds. Improved varieties are a major contributor of maize productivity growth. Recent literature associates the adoption of improved maize varieties in Ethiopia to a 47.6%–63.3% yield increase and consequently a 0.8–1.3 percentage reduction of poverty headcount ratio (Zeng et al., 2015).

Improved maize varieties can be categorized as either hybrid or open-pollinated improved varieties (OPVs). Hybrids have the highest yield, but are more costly to adopt as the restoration of hybrid vigor requires purchasing new seeds in each cropping season. The yields of OPVs are generally lower than those of hybrids (though still much higher than those of local varieties), but OPV seeds cost less than those of hybrids and may be recycled for up to three cropping seasons without significant yield loss. Many OPVs have specific traits which make their yields robust against challenging conditions such droughts and pests. Seed recyclability also makes them especially attractive for areas with underdeveloped seed markets (Jaleta et al., 2013).

Adoption of improved maize varieties varies across agro-ecological regions throughout Ethiopia (Jaleta et al., 2013). Our data suggest that adoption rates as measured by area are higher in places of higher maize potential. No single variety dominates the whole adoption scenario, but hybrids are more popular than OPVs in general.

## 3 Theoretical framework

To illustrate the potential relationship between land rental contracts and the adoption of improved crop varieties, we build a simple theoretical model below. Our model is comparable to the mainstream literature that links land ownership and agricultural technology adoption (e.g. Deininger and Jin, 2006), but extends it to differentiate cash-rental and sharecropping contracts and to capture the specific characteristics of productivity-enhancing technologies. In this model, each farmer i is categorized as an owner-operator (O), a cash-renter (H), or a sharecropper (S). Regardless of land ownership, farmer i maximizes the present value (${PV}_{i}$) of current cropping returns ($R_{i})$, net of total costs ($C_{i}$), plus the expected future net returns,$\;V_{i}$, consisting of all future revenues assumed to be realized in the second period and possibly downscaled by a tenure risk indicator, $\theta_{i}$ (${0\leq \theta }_{i}\leq$1), due to the risk of losing land use rights (with rdenoting the discount factor):

(1) $$\max{PV}_{i}=R_{i}-C_{i}+\theta_{i}\frac{V_{i}}{1+r}$$

The total cost, $C_{i}$, captures both production/technology inputs, $I_{i}$, and possible land cost, $L_{i}$, i.e. $C_{i}=I_{i}+L_{i}$. $R_{i}$ and $C_{i}$ can be functions of household characteristics, plot characteristics, and crop variety adoption decision. For illustrative purposes, we consider the adoption decision, $A_{i}$, as a continuous argument of both $R_{i}$ and $C_{i}$. $A_{i}$ also reasonably captures the effort level of farmer i in face of extra investment reflected in $C_{i}$. In reality, it can be either the acreage of improved crop varieties (measured in hectares) or the area share of improved crop varieties among the whole cultivated area (measured as a ratio between zero and one). Assume that both $R_{i}$ and $C_{i}$ are fully differentiable with respect to $A_{i}$ and satisfy standard properties that suggest the existence of a unique optimum.^2^ On the other hand, unlike other agricultural technologies that affect long-term productivity, $V_{i}$ is not likely to be affected by season-specific crop variety choices.^3^ For this reason, $V_{i}$ is assumed not to vary among farmer types, and so$\;V_{O}=V_{H}=V_{S}=V$. Eq. (1) can therefore be written as:

(2) $$\max{PV}_{i}=R_{i}(A_{i})-I_{i}(A_{i})-L_{i}(A_{i})+\theta_{i}\frac{V}{1+r}$$

Eq. (2) describes the problem for all types of land ownership considered in the analysis: owner-operator (O), cash-renter (H), or sharecropper (S). For the owner-operator, $L_{O}=0$ as he/she does not pay for the owned land, and $\theta_{i}=1$ as there is no risk of eviction from the operated land. The optimal adoption level ($A_{O}^{*}$) is derived according to the following first-order condition:

(3) $$A_{O}\left(\partial R_{O}/\partial A_{O}-\partial I_{O}/\partial A_{O}\right)=0$$

Eq. (3) satisfies complementary slackness: either $A_{O}>0$ and $\partial R_{O}/\partial A_{O}-\partial C_{O}/\partial A_{O}=0$, or $A_{O}=0$ and $\partial R_{O}/\partial A_{O}-\partial C_{O}/\partial A_{O}<0$.^4^

The case of the cash-renter is different as the land cost is no longer zero, i.e. $L_{H}>0$. However, since the land rent is fixed, it does not vary with the tenant’s adoption behavior, i.e. $\partial L_{H}/\partial A_{H}=0$. The tenure risk indicator, $\theta_{H}$, can take any value between zero and one, yet it is not likely affected by varietal adoption either since the rent is predetermined free of crop variety choices.^5^ Moreover, the expected future net returns are again not affected by adoption for reasons above. Therefore, the first-order condition for the cash-renter can be expressed using similar notation as:

(4) $$\partial R_{H}/\partial A_{H}-\partial I_{H}/\partial A_{H}=0$$

Therefore, the first-order condition for the cash-renter as well as the optimal level of adoption is exactly the same as that for the owner-operator. This leads to our first testable hypothesis:

Hypothesis 1 *Land rental does not affect the adoption decisions of cash-renters, who are as likely to adopt improved crop varieties as owner-operators.*

This result is intuitive because crop revenues are received after each cropping season, and therefore the cash-renter adopts improved crop varieties as long as they are profitable in the current period. The risk of eviction in the future simply does not play a role as it is not changeable by current adoption, though it can increase current and thus the total discounted profit over time,$\;{PV}_{H}$. This result differs from that for other agricultural technologies such as conservation strategies which produce future benefits. In the latter cases, land rental can preclude tenants from future technology-induced benefits through eviction and may discourage adoption (Soule et al., 2000).

The case of the sharecropper is more complicated than that of the cash-renter for two reasons. First, unlike either owner-operators or cash-renters who receive all revenues and bear all costs, a sharecropper assumes only a share of each and the shares can differ. Instead of paying a separate land rent, the sharecropper forgoes a proportion of the cropping revenue in exchange of land use rights. Second, although future revenues (V) are not likely altered by crop varieties adopted in the current period, the chance of continuing cultivation on the plot, again captured by the tenure risk indicator, can indeed be affected as a profit-driven landlord may choose to continue contracting with a sharecropper who generates higher returns for him/her through the adoption of improved crop varieties. Given these differences, we can write the sharecropper’s problem as:

(5) $$\max{PV}_{S}={\alpha R}_{S}(A_{S})-{\beta I}_{S}(A_{S})+\theta_{S}\left[\left(1-\alpha \right)R_{S}(A_{S})\right]∙\frac{V}{1+r}$$

where α is the revenue share (0<α<1); β is the cost share (0<β<1); $\theta_{S}$ is the tenure risk indicator (${0\leq \theta }_{S}\leq$1 due to the risk of eviction); and α≠β in general. The third term is the tenure-risk weighted, discounted expected future net returns received by the sharecropper. In this case, the land cost of the sharecropper is computed as the difference between the forgone revenue and the compensated input cost (shared by the landlord), i.e. ${(1-\alpha )R}_{S}(A_{S})-{(1-\beta )I}_{S}$, and therefore Eq. (5) describes a specific scenario derived from Eq. (2). Unlike the cash-renter case, $\theta_{S}$ is now affected by the landlord’s share of cropping revenue,$\;\left(1-\alpha \right)R_{S}$, where the total revenue ($R_{S}$) is further affected by the sharecropper’s adoption decision, $A_{S}$. The first-order condition can then be written as:

(6) $$\alpha \frac{\partial R_{S}}{\partial A_{S}}-\beta \frac{\partial I_{S}}{\partial A_{S}}+\left(1-\alpha \right)\frac{\partial \theta_{S}}{\partial R_{S}}∙\frac{\partial R_{S}}{\partial A_{S}}∙\frac{V}{1+r}=0$$

Eq. (5) intuitively suggests the equimarginal principle: at the optimum, the marginal benefit of improved crop variety adoption, including current marginal revenue plus future marginal revenue due to increased chance to cultivate the same land in the future, is equal to the marginal cost of adoption the sharecropper bears in the current period.

To derive the comparative statics of interest, i.e. the impact of sharecropping contract on improved crop variety adoption,$\;\partial A_{S}/\partial \theta_{S}$, we formally assume $\partial \theta_{S}/\partial R_{S}\geq 0$. It is intuitive that higher revenue in most cases may financially incentivize a landlord to continue the contract with a sharecropper. In the first (and rare) case where the landlord is profit-neutral and an interior solution exists, i.e.$\;\partial \theta_{S}/\partial R_{S}=0$, Eq. (5) reduces to:

(7) $$\alpha \left(\partial R_{S}/\partial A_{S}\right)-\beta \left(\partial I_{S}/\partial A_{S}\right)=0$$

Under the equal share rule that α=β, it is straightforward to see that the first-order condition and optimal level of adoption for the sharecropper is exactly the same as that for the owner-operator, as shown in Eq. (2). In the real world, however, departures from the equal share rule are also observed, where issues such as output uncertainty and input monitoring difficulty may result in contracts where α≠β (Braverman and Stiglitz, 1986). In these cases, a sharecropper can be either more or less likely to adopt improved crop varieties, but all these cases would be trivial in the real world as most landlords are profit-driven ($\partial \theta_{S}/\partial R_{S}>0$, the conventional rationality assumption).

As it is assumed in footnote^3^ that$\;\partial R_{S}/\partial A_{S}>0$, $\partial \theta_{S}/\partial R_{S}>0$ further implies that the last term of Eq. (5) is positive while the sum of first two terms is negative regardless of the values α and β take. With implicit function results, we finally derive Eq. (7) and our second testable hypothesis:

(8) $$\frac{\partial A_{S}}{\partial \theta_{S}}=\frac{\left(1-\alpha \right)V}{\left(1+r\right)\left[\alpha \left(\partial R_{S}/\partial A_{S}\right)-\beta \left(\partial I_{S}/\partial A_{S}\right)\right]}<0$$

Hypothesis 2 *Sharecroppers are more likely to adopt improved crop varieties than owner-operators given that these varieties are profitable and the landlord is profit-driven.*

This result is again intuitive: as long as improved crop varieties are profitable, a sharecropper will try to secure future profit flows through extension of the land rental contract by adopting improved crop varieties. As most landlords are profit-driven, and given the fact that sharecropping has been the predominant land rental contract type in Ethiopia (Holden et al., 2008), we further expect a positive overall impact of land rental on the adoption of improved crop varieties. Such overall impact is also evaluated in our empirical analysis after the assessment of contract-specific impacts of cash-rental and sharecropping.

## 4 Data description

The current analysis uses data from a household survey of Ethiopian maize farmers conducted during 2009–2010. Four regions were covered: Tigray, Amhara, Oromia, and Southern Nations, Nationalities, and People's Region (SNNPR), which together accounted for more than 93% of maize production in Ethiopia (Schneider and Anderson, 2010). The data were collected using a stratified random sampling strategy that appropriately accounted for the representativeness of areas with varying maize potential, and therefore are nationally representative for the maize growing areas.

The survey covered 1396 farm households from 124 villages (kebeles) in 30 districts (woredas) across the four regions, of whom 1359 households grew maize on 2496 plots during the surveyed period. Basic demographics, including characteristics of the household head, were reported at the household level. Total land and asset holdings, and infrastructure conditions such as distances (measured in walking minutes) to the nearest agricultural extension office (where most Ethiopian farmers buy improved crop seeds and meet extension personnel) and main market, were also reported at this level. Detailed maize cropping practices and physical conditions such as fertility, soil depth and slope were assessed by farmers at the plot level.

Land use rights (ownership), crop choice and technology adoption were reported at the plot level. Maize plots are classified as either “owned” or “rented in” (through either cash rental or sharecropping contracts). Further classification of the two types of land rental contracts, either cash-rental or sharecropping, is facilitated using the information of in-kind payments to land recorded in the crop utilization section of the survey. After intensive discussions with local experts, sharecropped plots are identified as those of positive in-kind payments to land of maize and cash-rented plots are those of zero in-kind payments to land. In our survey, crop utilization is recorded as crop- and season-specific rather than plot-specific, and differentiation of cash-rental and sharecropping would have been impossible if a household operated more than one rented plot. Fortunately, 46.4% of households operated only a single maize plot in the surveyed period and 85.53% of households who cultivated multiple maize plots owned all these plots. To help identify sharecropped plots, the magnitudes of in-kind payments are further checked with total plot-level maize outputs, and a few observations where in-kind payments exceed total output are identified as misreported and dropped. Finally, households with either unidentifiable contract types or missing values are dropped, and 2359 maize plots operated by 1300 households remain in our empirical analysis with all necessary information. To minimize concerns about sample selectivity, we further check if the characteristics of the excluded and included households and the plots they operated are similar, and find that they are.

Maize variety information was also reported at the plot level. Exact variety names recorded in the survey were classified as hybrid, OPV or local after detailed communication with breeding scientists in Ethiopia. Although both hybrids and OPVs are widely adopted, few plants are genetically pure in maize cropping practices as inbred lines are usually crossed through open pollination. Also, yields of hybrids (3,543 kilograms per hectare) and OPVs (3,068 kilograms per hectare) are similar in our data (with no statistical significance at 5% level through pairwise *t*-test). Therefore, varieties are only categorized as either improved (including both hybrids and OPVs) or local in our analysis. As suggested by local breeding scientists, any hybrid variety ever recycled or an OPV recycled for more than three seasons are categorized as local due to substantial productivity loss. Finally, the maize variety was unique for any plot in our data, and each plot is finally identified as having either an improved or a local maize variety.

Based on the binary categorization of maize varieties, our survey presents 541 non-adopters (who did not adopt any improved maize variety), 486 full adopters (who purely grew improved maize varieties) and 273 partial adopters (who grew both improved and local varieties on different plots). Household characteristics are reported in Table 1. Larger, wealthier land holders and those with more family members are more likely to adopt improved maize varieties. Adopting households, including both full and partial adopters, differ systematically from non-adopting households in that the latter are poorer and are more likely female-headed ones. Non-adopters also have smaller land holdings, live farther from main markets, and observe lower maize yields. Moreover, non-adopters are less likely to participate in land rental markets, through either cash-rental or sharecropping.

**Table 1:** Household Summary Statistics by Adoption Type.^a^

	Non-adopters^b^ (n = 541)	Full adopters^b^ (n = 486)	Partial adopters^b^ (n = 273)
Household size	6.26 (2.22)	6.53 (2.42)	6.91 (2.40)^**, †^
Total assets (ETB)^c^	13,474 (30,511)	18,982 (35,721)^**^	22,819 (61,171)^**^
Head age (years)	43.66 (12.61)	41.86 (13.00)^*^	43.30 (11.40)
Head gender (male = 1; female = 0)	0.92 (0.27)	0.95 (0.21)^*^	0.98 (0.15)^**, †^
Head marital status (married = 1; other = 0)	0.91 (0.29)	0.95 (0.22)^*^	0.96 (0.19)^**^
Head education (years)	2.54 (3.03)	2.94 (3.36)^*^	2.97 (3.13)
Main market distance (walking minutes)	114.5 (59.97)	83.60 (54.11)^**^	97.20 (60.52)^**, ††^
Extension office distance (walking minutes)	29.96 (30.50)	28.25 (27.78)	34.17 (35.02)^†^
Unmet credit need (yes = 1; no = 0)	0.03 (0.15)	0.03 (0.16)	0.02 (0.17)
Years living in village	37.21 (13.99)	36.55 (14.51)	37.78 (13.24)
Cooperation membership (yes = 1; no = 0)	22.55 (0.401)	23.67 (0.412)	23.34 (0.404)
Total land holding (ha)	2.10 (0.97)	2.13 (1.17)	2.37 (1.46)^**, †^
Total maize area (ha)	0.55 (0.54)	0.69 (0.64)^**^	1.09 (0.99)^**, ††^
Avg. maize plot area (ha)	0.37 (0.38)	0.45 (0.41)^**^	0.37 (0.28)^††^
Maize yield (kg/ha)	2170 (1,483)	3479 (2,176)^**^	2753 (1,380)^**, ††^
Cash-renter proportion (%)	4.62	5.76	7.69
Sharecropper proportion (%)	12.75	20.78	23.81

a Standard deviations are in parentheses.^*^ and ^**^ indicate the variable mean differs from that of non-adopters at 5% and 1% levels, respectively. ^†^ and ^††^ indicate the variable mean differs from that of adopters at 5% and 1% levels, respectively.

b Non-adopters are those who did not grow any improved maize variety in the survey period; full adopters are those who purely grew improved maize varieties; and partial adopters are those who simultaneously grew both improved and local varieties on different plots.

c Computed as the sum of reported current values of all itemized assets in Ethiopian Birrs (ETB). Daily average exchange rate in 2010 is 1 USD = 14.38 ETB.

Partial adoption of improved crop varieties widely exists in the developing world (e.g. Smale et al., 1994; Radhu et al., 2015; Kathage et al., 2016). In our data, partial adopters have the largest total landholding, wealth and total maize area with market access better than non-adopters but worse than full adopters (Table 1). The pattern that larger farms tend to be partial adopters is also observed in Radhu et al. (2015). While identification of the determinants of partial adoption is beyond the scope of the current study and is difficult with our cross-sectional observational data, literature generally suggests risk-averse farmers would partially adopt to reduce perceived downside production risk of improved varieties (Smale et al., 1994; Krishna et al., 2016), especially in the absence of formal insurance markets (Baumgärtner and Quaas, 2010). Therefore, it is primarily the intermediate smallholders that are fully incentivized to intensify crop production through full adoption of improved maize varieties (Radhu et al., 2015).

Among the 2359 maize plots operated by these 1300 households, 1965 were operated by owners, 89 by cash-renters and 305 by sharecroppers. Plot characteristics are reported in Table 2. Rented-in plots are farther from operators’ homes and sharecropped plots are generally larger and more fertile. Adoption rates are higher on rented plots. Sharecropped plots have slightly higher maize yields than owner-operated ones, yet such difference is only marginally significant and could largely result from the higher adoption rate of improved maize varieties with sharecropping. Finally, although “reverse tenancy” is common in Ethiopia (resource-poor landlords rent out land to resource-rich tenants in change of fixed income to hedge against production risks, ses Ghebru and Holden, 2008; Holden and Bezabih, 2008), our data show no evidence for either land holding or wealth differentials by land ownership.

**Table 2:** Plot Summary Statistics by Tenure Type.^a^

	Owned (n = 1965)	Cash-rented (n = 89)	Sharecropped (n = 305)
Plot size (ha)	0.39 (0.39)	0.41 (0.32)	0.49 (0.41)^**^
Soil fertility (fertile = 1; otherwise = 0)	0.08 (0.27)	0.09 (0.29)	0.20 (0.40)^**, †^
Soil slope (flat = 1; otherwise = 0)	0.67 (0.47)	0.63 (0.49)	0.67 (0.47)
Soil depth (deep = 1; otherwise = 0)	0.41 (0.49)	0.42 (0.50)	0.41 (0.49)
Distance from home (minute)	9.38 (16.96)	23.38 (31.29)^**^	24.28 (46.07)^**^
Cropping season (long = 1; short = 0)	0.93 (0.26)	0.97 (0.18)	0.91 (0.29)
Total household land holding (ha)	2.28 (1.42)	2.19 (1.16)	2.25 (1.40)
Total household asset (ETB)^b^	19,580 (43,564)	25,846 (79,201)	19,882 (67,475)
Maize yield (kg/ha)	2751 (2,024)	2614 (1,694)	2960 (2,029)
Adopting area proportion (%)^c^	38.34	41.79	55.93

a Standard deviations are in parentheses. ^*^ and ^**^ indicate the variable mean differs from that of owned plots at 5% and 1% levels, respectively. ^†^ and ^††^ indicate the variable mean differs from that of cash-rented plots at 5% and 1% levels, respectively.

b Computed as the sum of reported current values of all itemized assets in Ethiopian Birrs (ETB). Daily average exchange rate in 2010 is 1 USD = 14.38 ETB.

c Sampling weights are accounted for.

## 5 Empirical strategy

Our empirical analysis is implemented in three steps. First, we consider the contract-specific impacts of land rental on improved maize variety adoption and estimate separate impacts of cash-rental and sharecropping (“contract model” hereafter). To see possible overall impact regardless of contract types, we further apply a similar procedure using a binary land rental indicator without differentiation of contract type (“tenure model” hereafter). Multiple robustness exercises are finally performed to build confidence in the findings.

Empirical modeling is implemented at the plot level, as a farmer could operate both owned and rented-in plots at the same time. As discussed in our theoretical model above, for plot j of farmer i, both crop revenue ($R_{ij}$) and cost ($C_{ij}$) are assumed to be functions of household characteristics, $H_{i}$, plot characteristics, $P_{ij}$, adoption decision, $A_{ij}$, and unobservables, $\varepsilon_{ij}$. Use $T_{ij}$ to denote land tenure. Our empirical model with adoption as the dependent variable can be conceptually specified by solving the optimization problem for plot j of farmer i:

(9) $$A_{ij}=A_{ij}\left(H_{i},P_{ij},T_{ij},\varepsilon_{ij}\right)$$

where $\varepsilon_{ij}$ is the random disturbance that comes into play in the regression model. Specifically, the vector $H_{i}$ includes household size, total household wealth, total land holding the age, gender, marital status and education of the household head, the walking distances to the nearest main market and agricultural extension office, two social network indicators (number of years living in the village and farmers’ cooperative membership indicator) and a binary indicator suggesting if household i had unmet credit need in crop production during the surveyed year.^6^ The vector $P_{ij}$ includes plot size, soil fertility, soil slope, soil depth, distance from home and the cropping season. Although $T_{ij}$ is also a plot feature, we further set it apart from other plot characteristics as it is of our main interest. In the contract model, $T_{ij}$ is a vector of two binary indicators (of cash-rental and sharecropping) which take the value of one for rented plots and zero for owned plots. While in the tenure model, $T_{ij}$ is one binary indicator of land rental. $T_{ij}$is treated as endogenous given its choice nature as reflected by possible correlations with unobservables ($\varepsilon_{ij}$).

Econometric approaches to binary choice models with endogenous regressors include linear probability model with instrumental variable estimation, multivariate probit model with maximum likelihood estimation and control function probit model with two-stage estimation. Estimation of a linear probability model by two-stage least squares (2SLS) is suitable in our case as the causal impact of land rental on adoption is our main interest (Angrist, 2001). Although a nonlinear model may fit the conditional expectation function better for limited dependent variable models, the difference in terms of marginal effects is usually indistinguishable (Angrist and Pischke, 2009). A multivariate probit model with maximum likelihood estimation is an alternative, where the determinants of multiple correlated binary outcomes are jointly estimated. However, the multivariate probit model is more restrictive than the linear probability model as it relies on the assumption of joint normality of error terms that is usually violated. Moreover, it is vulnerable to incorrect first-stage specifications, but the 2SLS estimation of a linear probability model is robust against such misspecification (Angrist, 2001; Lewbel et al., 2012). Unlike either of these procedures, the control function probit model with two-stage estimation is designed for continuous endogenous variables (Rivers and Vuong, 1988), and our binary endogenous regressors would violate the distributional assumption of this approach. Therefore, we choose the linear probability model as our main estimation strategy, while we also estimate multivariate probit models, namely a trivariate probit contract model with three binary choices (adoption, cash-rental decision, sharecropping decision) and a bivariate probit tenure model with two binary choices (adoption, land rental decision), for comparison purposes.

Successful identification of our empirical models requires the availability of appropriate instruments, which should be correlated with land rental decision but not maize variety choice other than through land rental decision. Two instruments are included in our analysis: 1) the change of total land holding in the last five years, and 2) for each household, the proportion of smaller holders in the village who rented-in land during the study period. Both instruments are worth careful discussion regarding their validity.

The first instrument is computed as the acreage difference between household-level land holdings of the current period compared to that of five years ago. As land sales market is nonexistent in Ethiopia (Holden et al., 2008), change of land holdings mostly occurs through governmental land redistribution that is exogenous to the household (Deininger and Jin, 2006), except for land transfers through inheritance which should be rare in the short run. We posit that recent loss of land encourages land rental for a tenant, while recent gain of land discourages it. However, such change is unlikely to affect maize variety choices directly. Moreover, sufficient variation exists as 17.4% households (226 out of 1300) observed changes in land holdings.

The second instrument reflects the thickness of village land rental market. Based on within-village ranking of household land holdings, the proportion of households who own a smaller acreage of land as compared to each household is constructed.^7^ The renter proportion over smaller holders rather than of the whole village is employed given the notion that the smallness of land holding could stimulate land rental market participation to meet household food demand, and the instrumental variable construction in this way also allows within-village variation of the instrument among households. It is speculated that the village land rental markets are thicker and associated transaction costs are lower if more farmers rent in land. Hence, this instrument should directly affect land rental decision but not improved maize variety adoption. In fact, it is recently found that poorer and smaller holders may as likely adopt improved maize varieties as richer and larger holders in Ethiopia (Zeng et al., 2015). This leaves only one potential source of bias: the correlation between the position of a household in the within-village land holding ranking that may systematically affect the variation of the instrument and unobservable characteristics that may affect improved maize variety adoption, which we view as highly unlikely.^8^ Therefore, the exclusion restriction should again be satisfied.

## 6 Results

The contract model is first estimated. Results are presented in Table 3. Of our main interest is the linear probability model estimated via 2SLS, which appears to be appropriately identified according to test statistics. Moreover, the significant error correlation in the bivariate probit model suggests the simultaneity of improved maize variety adoption and land rental decisions. Therefore, it makes sense to draw inference based on the 2SLS estimates while using the bivariate probit results for robustness check purposes. We also estimate simple linear probability model and probit model without instrumentation in search of the sign of selection. Finally, standard errors in linear probability models are clustered at the district level, the primary sampling unit.

**Table 3:** Impact Estimates of Contract Model (n = 2359).^a^

	Linear probability models		Maximum likelihood procedures^b^	
	OLS	2SLS	Probit	Trivariate probit
Cash-rental	0.115 (0.054) ^*^	0.069 (0.055)	0.107 (0.048) ^*^	0.084 (0.055)
Sharecropping	0.304 (0.037) ^**^	0.142 (0.031) ^**^	0.344 (0.046) ^**^	0.159 (0.033) ^**^
Plot size	0.039 (0.008) ^**^	0.051 (0.015) ^**^	0.041 (0.011) ^**^	0.038 (0.007) ^**^
Soil fertility	−0.041 (0.028)	−0.028 (0.023)	−0.032 (0.027)	−0.019 (0.034)
Soil slope	0.032 (0.027)	0.032 (0.022)	0.027 (0.019)	0.040 (0.026)
Soil depth	0.069 (0.022) ^**^	0.074 (0.020) ^**^	0.063 (0.022) ^**^	0.066 (0.021) ^**^
Dist. from home	0.001 (0.000)	0.001 (0.000) ^**^	0.001 (0.001)	0.001 (0.001) ^*^
Cropping season	0.127 (0.035) ^**^	0.145 (0.038) ^**^	0.131 (0.042) ^**^	0.119 (0.042) ^**^
Household size	0.007 (0.005)	0.006 (0.006)	0.008 (0.005)	0.006 (0.005)
Total asset	−0.000 (0.000)	0.000 (0.000)	−0.000 (0.000)	−0.000 (0.000)
Head age	−0.002 (0.001)	−0.002 (0.001) ^*^	−0.002 (0.001)	−0.003 (0.001) ^**^
Head gender	0.051 (0.056)	0.065 (0.048)	0.052 (0.073)	0.074 (0.074)
Head marital status	0.036 (0.064)	0.044 (0.062)	0.066 (0.068)	0.051 (0.067)
Head education	0.001 (0.005)	0.003 (0.001) ^*^	0.001 (0.004)	0.004 (0.002) ^*^
Main market dist.	−0.002 (0.000) ^**^	−0.002 (0.000) ^**^	−0.002 (0.000) ^**^	−0.002 (0.000) ^**^
Extension office dist.	0.001 (0.001)	−0.001 (0.000)	−0.001 (0.000)	−0.001 (0.000)
Total land holding	−0.001 (0.000)	−0.001 (0.002)	−0.001 (0.001)	−0.001 (0.001)
Unmet credit need	−0.009 (0.004) ^*^	−0.014 (0.004) ^**^	−0.007 (0.004) ^*^	−0.009 (0.003) ^**^
Years living in village	0.007 (0.011)	0.004 (0.011)	0.014 (0.015)	0.004 (0.014)
Cooperation membership	0.022 (0.012)	0.017 (0.043)	0.023 (0.016)	0.012 (0.020)
Constant	0.192 (0.167)	0.112 (0.173)		
*F* statistic	16.11 ^**^	22.79 ^**^		
*F* test of 1st stage IV		47.41 ^**^		
Underidentification^c^		36.88 ^**^		
Weak identification^d^		27.26		
LR / Wald chi-square			293.05 ^**^	361.44 ^**^
*ρ*_adoption and cash-rental_				0.198 ^**^
*ρ*_adoption and sharecropping_				0.345 ^**^
*ρ*_cash-rental and sharecropping_				0.127 ^*^

a Dependent variable is the binary improved maize variety adoption indicator. Standard deviations are in parentheses. ^*^ and ^**^ indicate statistical significance at 5% and 1% levels, respectively.

b Marginal impacts are reported.

c Kleibergen-Paap rank Lagrange Multiplier statistic is reported.

d Kleibergen-Paap rank Wald *F* statistic is reported.

Sharecropping contracts significantly increases the probability of improved maize variety adoption by 0.142 among sharecroppers. Such change is very close to, though slightly smaller than, the trivariate-probit marginal effect of 0.159. On the other hand, the impact among cash-renters are much smaller and statistically insignificant, which is intuitive and accords with Hypothesis 1 above. As a comparison, the impact magnitudes estimated by both OLS and simple probit procedures are much larger and even significant for cash-renters, suggesting the existence of positive selection. In other words, farmers may intentionally rent in land to cultivate improved maize varieties. For sharecroppers, this is intuitive as maize plots under sharecropping contracts are more fertile and larger than owned ones (see Table 2), and it can be reasonably speculated that sharecroppers may realize both productivity and the economy of scale at higher levels.

Among the covariates, larger plots, deeper soil and better education of the household head are positively associated with adoption, while older age and longer distance from the nearest main market are negatively associated with adoption. Farmers tend to adopt improved maize varieties in the long rainy season (meher) rather than the short season (belg), which is a common practice as the time span of the long rainy season (from mid-June to mid-September) better meets the water needs of most maize varieties, and higher maize yields are usually reached in the former. Most of these effects, however, are relatively small.

Given the estimates above, the overall impact of land rental on improved maize variety adoption can be positive if it is sufficiently driven by the impact of sharecropped plots that consist of 77.41% of all rented plots. Therefore, we further estimate such overall impact in the tenure model with a single binary land rental indicator rather than separate dummies for cash-rental and sharecropping. Results are presented in Table 4. Land rental increases the probability of improved variety adoption on that plot by 0.135, which is highly significant. The bivariate probit model suggests a similar impact of 0.151. Moreover, as in the contract model, the impact magnitudes using OLS and simple probit procedures are much larger than those with instrumentation, again suggesting positive selection. Covariate coefficients in the tenure model bear similar patterns to those in the contract model. Likewise, few such effects compare in magnitudes to that of the land rental indicator.

**Table 4:** Coefficient Estimates of Tenure Model (n = 2359).^a^

	Linear probability models		Maximum likelihood procedures^b^	
	OLS	2SLS	Probit	Bivariate probit
Land rental	0.365 (0.142) ^**^	0.135 (0.033) ^**^	0.277 (0.063) ^**^	0.151 (0.038) ^**^
Plot size	0.033 (0.007) ^**^	0.040 (0.011) ^**^	0.044 (0.014) ^**^	0.044 (0.009) ^**^
Soil fertility	−0.037 (0.039)	−0.016 (0.023)	−0.012 (0.030)	−0.021 (0.041)
Soil slope	0.039 (0.020) ^*^	0.033 (0.021)	0.030 (0.025)	0.015 (0.025)
Soil depth	0.067 (0.019) ^**^	0.060 (0.017) ^**^	0.079 (0.020) ^**^	0.073 (0.022) ^**^
Dist. from home	0.000 (0.001)	0.001 (0.000)	0.000 (0.000)	0.001 (0.001)
Cropping season	0.122 (0.036) ^**^	0.141 (0.042) ^**^	0.154 (0.051) ^**^	0.136 (0.039) ^**^
Household size	0.012 (0.008)	0.006 (0.005)	0.002 (0.005)	0.006 (0.004)
Total asset	−0.000 (0.000)	0.000 (0.000)	−0.000 (0.000)	0.000 (0.000)
Head age	−0.001 (0.001)	−0.002 (0.001)	−0.002 (0.001) ^**^	−0.002 (0.001)
Head gender	0.062 (0.055)	0.033 (0.057)	0.075 (0.066)	0.096 (0.062)
Head marital status	0.041 (0.061)	0.050 (0.072)	0.070 (0.041)	0.050 (0.034)
Head education	0.002 (0.003)	0.003 (0.002) ^*^	0.004 (0.003)	0.003 (0.002) ^*^
Main market dist.	−0.002 (0.000) ^**^	−0.002 (0.000) ^**^	−0.002 (0.000) ^**^	−0.002 (0.000) ^**^
Extension office dist.	0.000 (0.000)	−0.001 (0.000)	−0.001 (0.000)	−0.000 (0.000)
Total land holding	−0.001 (0.001)	−0.001 (0.001)	−0.001 (0.001)	−0.001 (0.001)
Unmet credit need	−0.019 (0.003) ^**^	−0.014 (0.003) ^**^	−0.008 (0.003) ^*^	−0.009 (0.001) ^**^
Years living in village	0.008 (0.005)	0.005 (0.014)	0.013 (0.020)	0.004 (0.014)
Cooperation membership	0.012 (0.015)	0.012 (0.042)	0.021 (0.018)	0.003 (0.022)
Constant	0.127 (0.148)	0.189 (0.095) ^**^		
*F* statistic	16.81 ^**^	16.33 ^**^		
*F* test of 1st stage IV		79.19 ^**^		
Underidentification^c^		64.95 ^**^		
Weak identification^d^		54.11		
LR / Wald chi-square			286.26 ^**^	536.60 ^**^
*ρ*_adoption and land rental_				0.213 ^**^

a Dependent variable is the binary improved maize variety adoption indicator. Standard deviations are in parentheses. ^*^ and ^**^ indicate statistical significance at 5% and 1% levels, respectively.

b Marginal effects are reported.

c Kleibergen-Paap rank LM statistic is reported.

d Kleibergen-Paap rank Wald F statistic is reported.

These results provide consistent and fairly strong evidence for our main hypotheses in Hypotheses 1 and 2: land rental does not affect crop variety adoption decisions of cash-renters but encourages it for sharecroppers. Moreover, a positive overall impact is found as the majority of rented plots were operated under sharecropped contracts. The impact magnitudes of land rental are larger than most covariate coefficients, suggesting the substantial explanatory power of this factor in crop variety adoption decision. Therefore, it is towards the robustness of these impact estimates that we now turn.

We implement several robustness check procedures in addition to the multivariate probit model estimation. The first two procedures address model specification issues. First, it is arguable that village-level unobservable heterogeneity may possibly exist, which could arise from local land rental market conditions, distinctive agroecological environments, different agricultural production practices, or varying social norms across localities. To check if this possibility threatens our main results, we control for village fixed effects and re-estimate all models. As seen from the first panel of Table 5, these new estimates are very close to our main estimates in all specifications.

**Table 5:** Impact Estimates via Robustness Check Procedures.^a^

	Linear probability models		Multivariate probit^b^	
	Contract model	Tenure model	Contract model	Tenure model
Instrumental variable estimation with village fixed effects (n = 2359)				
Cash-rental	0.068 (0.045)		0.062 (0.033)	
Sharecropping	0.139 (0.047) ^**^		0.160 (0.054) ^**^	
Land ownership		0.121 (0.028) ^**^		0.137 (0.059) ^*^

Instrumental variable estimation with yield risk measures (n = 2359)				
Cash-rental	0.071 (0.052)		0.069 (0.054)	
Sharecropping	0.097 (0.035) ^**^		0.134 (0.042) ^**^	
Land ownership		0.115 (0.039) ^**^		0.122 (0.036) ^**^

Instrumental variable estimation using long-season subsample (n = 2187)				
Cash-rental	0.081 (0.057)		0.065 (0.046)	
Sharecropping	0.164 (0.030) ^**^		0.185 (0.033) ^**^	
Land ownership		0.142 (0.035) ^**^		0.162 (0.041) ^**^

Ordinary least square estimation using partial-adopter subsample (n = 743)				
Cash-rental	0.207 (0.141)			
Sharecropping	0.228 (0.065) ^**^			
Land ownership		0.205 (0.064) ^**^		

a Dependent variable in the upper and central panel models is the binary improved maize variety adoption indicator, while in the lower panel it is the demeaned adoption indicator. Standard deviations are in parentheses. ^*^ and ^**^ indicate statistical significance at 5% and 1% levels, respectively.

b Marginal effects are reported.

The second procedure regards production risks that could affect improved maize variety adoption. One limitation of our cross-sectional data is the lack of *ex ante* yield statistics (existent before the cropping season) that could factor into crop variety choice made at the beginning of that season. Still, there is a need to detect any potential effect from production uncertainty, which could confound the impacts of land rental that is of our primary interest. This is more likely the case for sharecroppers who, unlike cash-renters, do not individually bare production risks. To test this, we have to refer to the *ex post* yield statistics at the district level and assume that the yield distribution realized after the surveyed season is representative of the real yield variations. This is a reasonable assumption as 2009–2010 was a fairly good cropping year according to climate statistics we obtained from the National Meteorology Agency of Ethiopia. Specifically, we consider the first two moments of yield, the mean and variance, both computed at the district level using plot-level measures. Impact estimates with these additional covariates are reported in the second panel of Table 5, which tend to be slightly smaller yet in no cases do they lose statistical significance. Since our inference is mainly qualitative, this again supports our main results.

We further implement two subsample analysis to test our main results. One concern may arise from cropping season. If land rental market conditions vary systematically with cropping season, participation in this market would be affected and our impact estimates could be inconsistent. This, however, is unlikely to be the case as Ethiopian farmers mainly cultivate maize in the long rainy season for reasons discussed above. As a result, only 172 of 2359 plots were cultivated in the short rainy season in our data, and related variation is rather limited. Nevertheless, we re-estimate all specifications with a homogenized subsample that only includes 2187 plots cultivated in the long rainy season to check if our main impact estimates are robust. As reported in the third panel of Table 5, the new estimates are slightly larger than our main results, suggesting that the latter are rather conservative. Therefore, this concern should be minimized.

As a final robustness check, we make use of a unique subsample of our survey data: 743 maize plots cultivated by 273 partial-adopters who grew both improved and local maize varieties on different plots in the surveyed period. Specifically, we consider a first-difference type model where the plot-level maize variety adoption decision is first demeaned within the household, and then regressed against both demeaned plot characteristics and demeaned land rental indicator(s). Household characteristics would drop out as there is no within-household variation. The model as linearized from Eq. (8) can be expressed as:

(10) $$\overset{-}{A_{ij}}=\beta_{1}\overset{-}{P_{ij}}+\beta_{2}\overset{-}{T_{ij}}+u_{ij\;}$$

Eq. (9) cancels out both observed and unobserved household-level heterogeneity. With further control for plot-level heterogeneity, the impact of land rental is captured by the estimate of$\;\beta_{2}$, which can be unbiasedly and consistently recovered through OLS estimation. As shown in the bottom panel of Table 5, the impact estimates for both cash-rented and sharecropped plots appear to be much larger than the full-sample 2SLS results. This provides strong evidence for the observed positive selection that farmers intentionally rent in plots to cultivate improved maize varieties. All these results suggest the robustness of our main impact estimates.

## 7 Concluding remarks

We assess the impact of land rental as manifested in two major contracts on improved crop variety adoption. While many previous studies hypothesize that the lack of land ownership discourages agricultural technology adoption (Gavian and Fafchamps, 1996; Abdulai et al., 2011; Oostendorp and Zaal, 2012), this is not the case for improved crop varieties. The theoretical model shows that land rental does not affect crop variety adoption of cash-renters, but encourages adoption by sharecroppers. Empirical analysis of Ethiopian maize farmers provides robust evidence in support of these hypotheses. As the benefits of improved crop varieties are widely documented in literature and their adoption is widely proposed as a potential means of welfare improvement in SSA, it is necessary to reevaluate the role of land rental markets as a means of stimulating improved crop variety adoption, which makes the current study policy-relevant.

Although our findings contradict many previous results concerning land tenure and agricultural technology adoption, they are not inconsistent with arguments for tenure security in SSA, which has been shown to be associated with multidimensional welfare improvements Kassie et al., 2011; Shiferaw et al., 2014; Zeng et al., 2015). In fact, policy implications would directly arise from the theory of second best, which justifies governmental intervention in related markets to offset the efficiency loss in a primary market that observes irremovable distortion (Lipsey and Lancaster, 1956). In our context, as land sales market is not legal in Ethiopia (and incomplete in most SSA countries, see Holden et al., 2008), improvements in the land rental market instead could substantially reduce the gap between land demand and supply and improve household welfare through improved crop variety adoption. It is recently found that land rental markets in Kenya promote farm productivity and significantly raise the incomes of land-constrained farm households (Jin and Jayne, 2013). Therefore, potential policies that ease transactions in local land rental markets may complement existing strategies to stimulate improved crop variety adoption. For example, agricultural extension services that promote information exchange in land rental markets, such as a village information board, may immediately reduce the search costs of both tenants and landlords. On the other hand, government actions to reduce policy uncertainty in land rental markets may potentially help formalize these markets to allow longer term contractual arrangements and incentivize farmers to participate. Although our results do not speak directly to the merits of these strategies, they are still worth policy consideration as a possible means to improve farm household welfare in SSA.

A first assessment of the impact land rental on improved crop variety adoption, our study has several limitations. The cross-sectional data do not support the investigation of the dynamic impacts associated with contractual length and/or renewal frequency. Also, limited data variation regarding other productivity-enhancing technologies, especially fertilizers, does not allow the modeling of joint adoption decisions. These open questions are worth further investigation with available data. At the minimum, our partial-equilibrium analysis does confirm the overall positive impact of land rental on crop variety adoption. Empirical results imply that improvements of land rental markets could potentially improve household welfare through participation in this market and associated profit-maximizing crop choices given the prominent role of agriculture in small household income generation in Ethiopia and other SSA economies.

## Footnotes

1 Land rental contracts can be either written or oral, and it is often the latter in rural Ethiopia (Holden et al., 2008).

2 For the rest of the analysis, it is only necessary to explicitly assume that$\;\partial R_{i}/\partial A_{i}>0$, i.e. adoption of improved crop varieties increases revenue with higher yields. It is trivially satisfied for most improved crop varieties.

3 This argument could be challenged if improved maize varieties are adopted with other technologies, particularly fertilizer as the residual of fertilizer might go to the next season. In our survey, fertilizer application is very common (utilized by more than 90% of surveyed farmers), and so its association with improved maize variety adoption is not strong. Including fertilizer use is therefore less informative given its limited variation and is problematic due to endogeneity and associated bias. Therefore, we choose to exclude fertilizer use in our empirical modeling.

4 For the rest of the analysis, we shall focus on interior solutions to derive comparative statics. Obviously, some operators would not adopt improved crop varieties for a number of reasons regardless of the tenure type (owner-operator, cash-renter and sharecropper), yet identification of those constraints are beyond the scope of the current study. Therefore, it is of our primary interest to see how tenure arrangements would affect the level of improved variety adoption which, for illustrative purposes, could be better captured by comparative statics assuming an interior solution.

5 It could be argued that the profitability and increasing adoption of improved maize varieties might increase cash rents over time. If that is the case, a positive correlation would be expected between cash-rental and maize variety adoption. Empirical investigation of this, however, would require multiple rounds of observations and is not supported by our data. Nevertheless, the current approach does not lose generality in the short-term investigation of the hypothesized impacts.

6 This measure is constructed using two survey questions: 1) whether a household had credit need in crop production in the surveyed year, and 2) whether such need was eventually met. Some farmers might not need credit, while some others in need of credit might have successfully secured it. Hence, this measure suggests whether access to credit was a “real” constraint in maize production during the surveyed period.

7 To further illustrate our instrumental variable construction, consider a village where 12 households were randomly surveyed. For a household whose total land holding ranked 4^th^ over these 12 households and who observed 3 renters (either cash-renter or sharecropper) among the 8 smaller holders, the instrument takes the value of 3/8=0.375.

8 The validity of this instrument would be threatened, for example, if more capable farmers (with higher ability, more experience, etc., which could affect improved maize variety adoption) would be able to expand his/her land holding over time (which therefore would change his/her within-village land holding ranking). However, as land sales market is absent in Ethiopia where the only sources of acreage change are the rare events of governmental land redistribution and land inheritance, such concern should be minimized. Therefore, the validity of both instruments rely critically on the plausible exogeneity of land holding change, a special feature of the Ethiopian land market.
